# Hybrid Signal Processing Technique to Improve the Defect Estimation in Ultrasonic Non-Destructive Testing of Composite Structures

**DOI:** 10.3390/s17122858

**Published:** 2017-12-09

**Authors:** Kumar Anubhav Tiwari, Renaldas Raisutis, Vykintas Samaitis

**Affiliations:** Prof. K.Baršauskas Ultrasound Research Institute, Kaunas University of Technology, K. Baršausko St. 59, LT-51423 Kaunas, Lithuania; renaldas.raisutis@ktu.lt (R.R.); vykintas.samaitis@ktu.lt (V.S.)

**Keywords:** signal processing, ultrasonic, non-destructive testing, composite, guided waves, GFRP, wind turbine blade, cross-correlation, wavelet transform, Hilbert–Huang transform

## Abstract

This work proposes a novel hybrid signal processing technique to extract information on disbond-type defects from a single B-scan in the process of non-destructive testing (NDT) of glass fiber reinforced plastic (GFRP) material using ultrasonic guided waves (GW). The selected GFRP sample has been a segment of wind turbine blade, which possessed an aerodynamic shape. Two disbond type defects having diameters of 15 mm and 25 mm were artificially constructed on its trailing edge. The experiment has been performed using the low-frequency ultrasonic system developed at the Ultrasound Institute of Kaunas University of Technology and only one side of the sample was accessed. A special configuration of the transmitting and receiving transducers fixed on a movable panel with a separation distance of 50 mm was proposed for recording the ultrasonic guided wave signals at each one-millimeter step along the scanning distance up to 500 mm. Finally, the hybrid signal processing technique comprising the valuable features of the three most promising signal processing techniques: cross-correlation, wavelet transform, and Hilbert–Huang transform has been applied to the received signals for the extraction of defects information from a single B-scan image. The wavelet transform and cross-correlation techniques have been combined in order to extract the approximated size and location of the defects and measurements of time delays. Thereafter, Hilbert–Huang transform has been applied to the wavelet transformed signal to compare the variation of instantaneous frequencies and instantaneous amplitudes of the defect-free and defective signals.

## 1. Introduction

Composite materials, in comparison to metals, have specific features such as lightweight, higher tensile and compressive strengths, higher resistance to corrosive effects, high stiffness, and low density [1]. That is why the composite materials such as carbon fiber reinforced plastics (CFRP) and glass fiber reinforced plastics (GFRP) are widely used to manufacture components for the applications under varying loads, including aircraft wings and wind turbine blades [2]. Although composites inherit all valuable features of their individual components, failure or defects can occur during a manufacturing process or when in service. Manufacturing defects can be caused by flaws, incorrect fiber volume and misalignment of ply. On the other hand, some defects such as bonding failures, cracks, fractures, or damage caused by moisture may arise during aircraft service [3,4]. It has been estimated that the total expenses including maintenance, inspection, and structural health monitoring of an aircraft covers 25% of its life cycle cost [5,6].

Composite materials have additional problems, such as high structural noise and acoustic attenuation as compared to its metallic counterpart, due to their multi-layered structure which adds more complexity to the process of locating and sizing the defects [7]. There are various non-destructive testing (NDT) methods—such as radiographic testing, ultrasonic testing, electromagnetic testing, etc.—which may be used in order to identify defects in composite materials. The selection of an appropriate method depends on the property of the material and the nature of the defect. Due to high sensitivity and volumetric nature, ultrasonic guided wave (GW) testing among all available NDT methods has been extensively used for inspection of these materials in order to estimate the size and location of defects [8,9,10,11].

**Figure 1:**
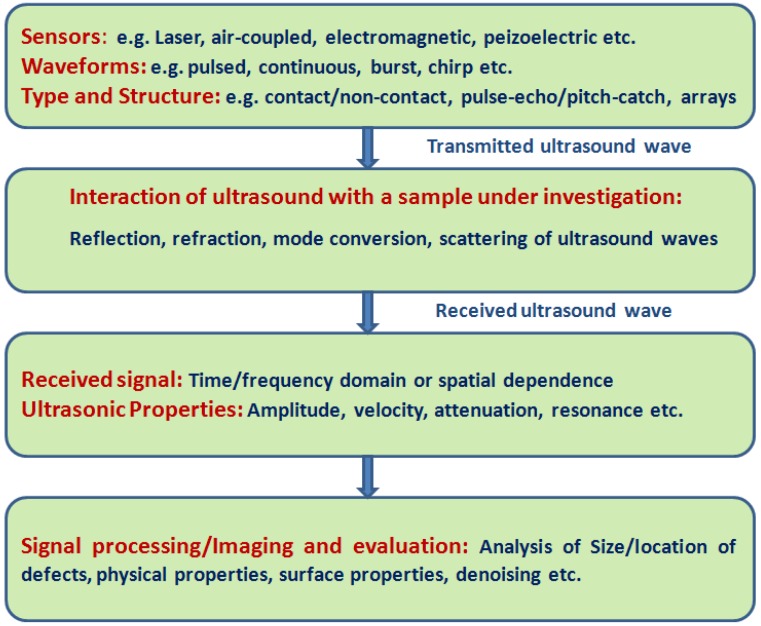
The basic structure of ultrasonic measurement system.

The principle of ultrasonic testing is based on detection and analysis of received ultrasonic waves [12]. The basic structure of ultrasonic measurement system with a description of signals, parameters, and properties is shown in Figure 1.

Many ultrasonic sensors or transducers such as laser, electromagnetic, air-coupled transducers etc. have been developed for the testing of fiber and metal based composite materials [13,14,15,16,17]. The contact and non-contact ultrasonic methods are available for testing of materials, with some merits and demerits, and effectiveness of usage depends on operating conditions and limitations of their applicability. Although ultrasonic NDT is an efficient technique for defect estimation in composites, the non-homogeneous structure of composite materials makes it complicated, and high attenuation of the ultrasonic signal may occur [18]. It was observed that the interaction of ultrasonic guided waves with a composite structure (e.g., wind turbine blade or aircraft wing), depends on many factors such as frequency of excitation, shape and geometry of the structure, material properties and propagation direction, etc. [19,20,21]. Some possible phenomena—such as scattering of GW, reflection, refraction, or mode conversion—occur during interaction of ultrasonic GW with the structure. From various characteristics of the received signal—such as time of flight, amplitude, etc.—information about damage in the inspected structure can be obtained by applying the appropriate signal processing approach.

The signal processing on the experimentally acquired ultrasonic signals depends on visualization of the signal. The C-scan image is most common in terms of visualization of defects, which can be obtained by integrating the limited information of several B-scan images. Various algorithms had already been proposed for the estimation of defects using the 3D reconstruction of C-scan or series of B-scan data [22,23,24]. Although a single B-scan image is not enough to locate and size defects effectively, still, adopting the proper signal processing can improve the accuracy of the analysis. In this research, the various signal processing methods were combined to estimate the disbond type defects from a single B-scan. This research work is the extended version of previous research, published in the conference proceedings [25]. Instead of different signal processing techniques applied separately on the experimentally obtained data in the previous research, this time, the hybrid signal processing technique is proposed and demonstrated in detail. This clearly improves defect analysis and visualization informativeness of results.

The objective of the present research was the development of a hybrid (mixed) signal processing technique to improve the estimation of the disbond type defects with diameters of 15 mm and 25 mm during the investigation of a segment of wind turbine blade (WTB) by ultrasonic guided waves (GW). The segment of WTB was constructed from GFRP material and defects were presented on the trailing edge of the sample. The milling (mechanical machining) process was used to create disbond type defects. The low-frequency (LF) ultrasonic system developed by the Ultrasound Research Institute, Kaunas University of Technology, was used in the experimental analysis. The proposed hybrid signal processing technique combined the features of three ultrasonic signal processing methods—cross-correlation, wavelet transform (WT) and Hilbert–Huang transform (HHT)—in order to extract the defect information from a single B-scan.

The main contributions of the research can be summarized as follows:

- The contact-type ultrasonic transmitter–receiver system fixed on a moving panel was developed to acquire the A-scans at each scanning step of 1 mm up to the overall scanning distance of 500 mm.
- The novel hybrid signal processing technique was proposed to extract the defect information from a single B-scan image.
- The WT with 8 level-Daubechies wavelets (db-16) and cross-correlation were applied on experimental data in order to size and locate the defects and estimate time delays between the arrival times of defect-free and defective signals.
- The instantaneous characteristics of the defect-free and defective signals were compared using the HHT.

The organization of the article is as follows. Section 2 describes the selected ultrasonic signal processing techniques which can be applied to ultrasonic NDT of composites. The experimental analysis is explained in Section 3 and the hybrid signal technique combining the cross-correlation, wavelet transform (WT), and Hilbert–Huang transform (HHT) is proposed in Section 4. The results and discussions are expressed in Section 5. Finally, the conclusive remarks are summed up in Section 6.

## 2. Ultrasonic Signal Processing

The major limitation in the ultrasonic NDT is the unavailability of suitable signal processing methods for the processing of noisy ultrasonic signals in order to estimate the flaws/defects on the structure in an effective manner [26]. The signal processing techniques are required not only for the analysis of received ultrasonic signals but also the automation of these techniques can further enhance the reliability and repeatability of the non-destructive testing and evaluation (NDT&E) process [27,28].

The ultrasonic signal processing is performed in two subtasks: denoising the received ultrasonic signal and detection of the faults/defects using parameter estimation [29]. The denoising of the signal increases the signal-to-noise ratio (SNR) and thereafter defect/flaws information is extracted using the suitable parametric estimation. The various signal processing techniques are available to improve the accuracy of the defect estimation and characterization in ultrasonic NDT testing. These can be categorized as cross-correlation, Hilbert transform (HT), autoregressive analysis, split spectrum processing (SSP), wavelet transform (WT), Hilbert–Huang transform (HHT), etc. [30,31,32,33,34,35,36]. Each method can work either in time domain or frequency domain or in both the time and frequency domains. There are a few merits and limitations associated with each of the mentioned signal processing methods. Due to the structural noise, high attenuation and wave scattering in the application of ultrasonic NDT of composite structures, it is necessary to choose the appropriate method from the available ones or develop a new signal processing technique as per requirement. In this research, the emphasis was focussed on cross-correlation, WT, and HHT methods in order to extract the disbond type defects present in the GFRP sample.

### 2.1. Cross-Correlation Technique

Although having a number of iteration of multiplications and summations, the cross-correlation is still one of the simplest signal process techniques in order to compare the defect-free and defective signals. The cross-correlation of two signals depicts the degree of similarity between them. If the two signals show the greatest similarity, the output of the cross-correlation operation will correspond to that point to the extent maximum possible and vice versa. This technique could be used to compare the received signal with the reference signal and could possibly extract the information from dispersive wave modes or a change in signal waveform due to delay and scattering. If $x_{1}[n]$ and $x_{2}[n]$ are two discrete signals, the cross-correlation function C[n] will be defined by [37]

(1) $$C[n]=\sum_{k=-\infty }^{\infty }x_{1}[k]\cdot x_{2}[k+n]$$

The delay time between the reference and test signals can also be estimated by finding the peak of correlation. The digital windowing can also be combined with correlation in order to estimate the time-delays between the windowed signals [38]. The reference signal is not necessarily the transmitted signal. For the analysis of defects, the selected A-scan signal in the defect-free region can be compared to each A-scan signal of the generated B-scan along the scanning distance.

### 2.2. Wavelet Transform (WT)

One of the most widely used methods for suppression of noise from the ultrasonic signals in order to improve accuracy of defect estimation is the discrete wavelet transform (DWT) [39,40,41]. The basic principle is decomposition of signals into the elementary signals which are called wavelets. Each wavelet coefficient contains a signal and noise in the time–frequency domain. By manipulating the wavelet coefficients, the noise effect can be reduced. The process of DWT is based on the analysis of the signal at different scales by using different type filters. The high pass filters (HPF) are used for analyzing the high frequencies and low pass filters (LPF) are used to analyze the low frequencies. Hence, there are two functions performed by DWT which are a scaling function with low pass filtering and a wavelet function with high pass filtering.

Considering a signal *x*[*n*] is passing through a half-band HPF $h_{hp}[n]$ and an LPF $h_{lp}[n]$, according to Nyquist criterion, the filtering process eliminates the half of samples and initiates the level-1 decomposition. If the response of the HPF and LPF are $y_{h}[k]$ and $y_{l}[k]$ respectively, after sub-sampling by 2, it can be mathematically expressed as [42]

(2) $$y_{h}[k]=\sum_{n}x[n]\cdot h_{hp}[2k-n]$$

(3) $$y_{l}[k]=\sum_{n}x[n]\cdot h_{lp}[2k-n]$$

This procedure leads to halving the time resolution but doubling the frequency resolution. This process is repeated for further decomposition. After starting from the last level of decomposition, the concatenation of all coefficients leads to forming the DWT. The low-frequency components will have low amplitudes in the DWT and therefore can be removed in order to get a reduced signal.

In the process of wavelet denoising, the overall signal shape is preserved after lowering the components with smaller amplitudes without considering frequency in the account. Since the wavelet transform is based on the similarities between the signal and wavelet function, the higher amplitude of the decomposed signal is achieved if the correlation of signal and wavelet function is higher. Hence, the dispersive or distorted pulses of the signal are reduced.

The most promising way for selecting and discarding the wavelet coefficients is by using the soft or hard threshold method [43,44]. This approach leads to removing all wavelet coefficients with magnitude lower than the threshold and the rest are used for reconstruction of the signal. However, this way is not efficient in the case of correlated noise especially when dealing with composite materials such as GFRP. The soft thresholding rule by using the universal threshold is the best way to deal with signals with correlated noise [45]. Although wavelet transform is being used quite intensively in the field of ultrasonic signal processing, still there is a lack of basic principles for selection of various wavelet methods and performance of available special filters such as Daubechies, Coiflet, etc.

### 2.3. Hilbert Huang Transform (HHT)

The HHT was proposed by Huang et al. for the analysis and processing of non-stationary signals [46,47,48]. It is a combination of two signal processing techniques: Hilbert transform (HT) and empirical mode decomposition (EMD) or ensemble empirical mode decomposition (EEMD) [47,49,50,51]. First, the EMD/EEMD decomposes the nonlinear and non-stationary signal into various linear and stationary intrinsic mode functions (IMFs), and then individual frequencies are calculated using HT. The decomposed ultrasonic signal can be represented as the local energy or amplitude distribution in the time-frequency plane which is called the Hilbert–Huang spectrum. This distribution differs in the case of defect-free and defected regions which in turn enable to extract the information about the defects for further processing. As compared to the Fourier transform (FT) and wavelet transform (WT), the HHT is adaptive and can be used for non-linear and transient signals. Various research analyses suggest that HHT using the EEMD decomposition is among the best signal processing methods in the NDT testing of composites [52,53]. In this work, EEMD was used instead of EMD for the decomposition of defective and defect-free signals and then HT was used to extract the instantaneous characteristics of the signals.

#### 2.3.1. Ensemble Empirical Mode Decomposition (EEMD)

There are various decomposition techniques for the multicomponent signals and EMD and EEMD are the most popular [47,49,50,51]. Both of these techniques are used to decompose the signals in IMFs or modes in order to analyze the intrinsic properties. For an individual signal component to be an IMF, two conditions must be satisfied:

- The extremum numbers in the signal must be either equal to the number of zero-crossings or differ by one.
- Zero mean value of lower and upper envelope.

EMD is an efficient decomposition method for analysis of nonlinear and non-stationary signals [47]. However, there are some serious limitations associated with EMD, such as mode mixing, which is due to the oscillations of various amplitudes in an IMF or similar type of oscillations present in the different IMFs. The other limitation in EMD is called end effect, which arises during the spline fitting process to generate the envelope of the signal [54]. During this process, the big swing of the signal envelope could interfere and disturb the signal itself. The modified and improved version of EMD is called EEMD which overcomes the above-mentioned limitations of EMD by adding white noise to the signal. The white noise distributes itself uniformly in the time-frequency space which facilitates the signal bits of different scales to form onto the reference scales configured by the white noise [51].

The entire process of EEMD can be described in the following steps:

Step 1: If x(t) is an original signal, the new signal $x_{new}(t)$ will be generated by adding the white noise $n_{T}(t)$.

(4) $$x_{new}(t)=x(t)+n_{T}(t)$$

Step 2: After finding the local maxima and minima of $x_{new}(t)$, upper envelope $e_{\max}(t)$ and lower envelope $e_{\min}(t)$ are generated using the cubic spline interpolation method. The mean value e(t) will be calculated as

(5) $$e(t)=\frac{e_{\max}\left(t\right)+e_{\min}(t)}{2}$$

Step 3: The difference d(t) between the signal $x_{new}(t)$ and mean value e(t) is calculated as

(6) $$d(t)=x_{new}(t)-e(t)$$

Step 4: The stopping criterion will be fulfilled if d(t) follows the IMF definition. Then d(t) will be the ith IMF and will be denoted as $c_{i}(t)$. The residual $r_{i}(t)$ will replace the signal $x_{\mathrm{new}}(t)$ as

(7) $$r_{i}(t)=x_{new}(t)-c_{i}(t)$$

The $r_{i}(t)$ will be the new data set and the shifting process will again be performed to get further IMFs.

Step 5: If the criterion of IMF is not fulfilled, the x(t) in step 1 will be replaced with d(t) and the process from (ii) to (v) will be repeated until IMF is extracted and the monotonic residue is left.

Step 6: As long as the number of trials T is lower than ensembles E(T<E), T will be changed by T+1 in step 1. Hence, the new white noise will be generated and all the process will be repeated again.

Step 7: Finally, the ensemble mean of each IMF $\bar{c_{k}}$ and corresponding residue $\bar{r}$ are calculated as

(8) $$\bar{c_{k}}=\frac{1}{E}\sum_{T=1}^{E}c_{k,T}$$

(9) $$\bar{r}=\frac{1}{E}\sum_{T=1}^{E}r_{T}$$

The sum of all IMFs and the residue lead to reconstructing the original signal as

(10) $$x(t)=\sum_{i=1}^{n}\bar{c_{i}}+\bar{r}$$

The EEMD overcomes the deficiencies of EMD but the reconstructed signal using EEMD may still include the residual noise, and different modes may be produced with different realizations of signals. In order to produce the better reconstruction and separation of modes, EEMD method with adaptive noise was used in this research [55].

#### 2.3.2. Hilbert Transform (HT) and Instantaneous Characteristics

After decomposing of the original signal into the IMFs, the IMFs of interest can be selected and summed up in order to reconstruct the new filtered time signal y(t) for further processing. The instantaneous characteristics of this signal can be calculated using the HT [56].

The HT of a real-valued signal y(t), $y_{h}(t)$ is defined as

(11) $$y_{h}(t)=\frac{1}{\pi }\int_{-\infty }^{\infty }\frac{y(\tau )}{t-\tau }d\tau$$

The analytical signal z(t) of the real-valued signal y(t) can be expressed as

(12) $$z(t)=y(t)+iy_{h}(t)=A_{inst}(t)\cdot e^{i\phi_{inst}(t)}$$

The instantaneous amplitude $A_{inst}(t)$ of the signal y(t) is the time-varying envelope of z(t) and can be expressed as

(13) $$A_{inst}(t)=\left|y(t)+iy_{h}(t)\right|$$

The instantaneous phase $\phi_{inst}(t)$ and instantaneous frequency $f_{inst}(t)$ are given as

(14) $$\phi_{inst}(t)=\tan^{-1}\frac{y_{h}(t)}{y(t)}$$

(15) $$f_{inst}(t)=\frac{1}{2\pi }\frac{d\phi (t)}{dt}$$

The HT has the property of preserving the signal domain which is used to present the signal energy variation with time. There are some limitations related to an instantaneous frequency such as its physical significance, defined only for monocomponent signals [57]. There may also be a possibility that the analytical signal cannot be defined, which in turn prevents the calculation of instantaneous frequency.

## 3. Experimental Investigation

**Figure 2:**
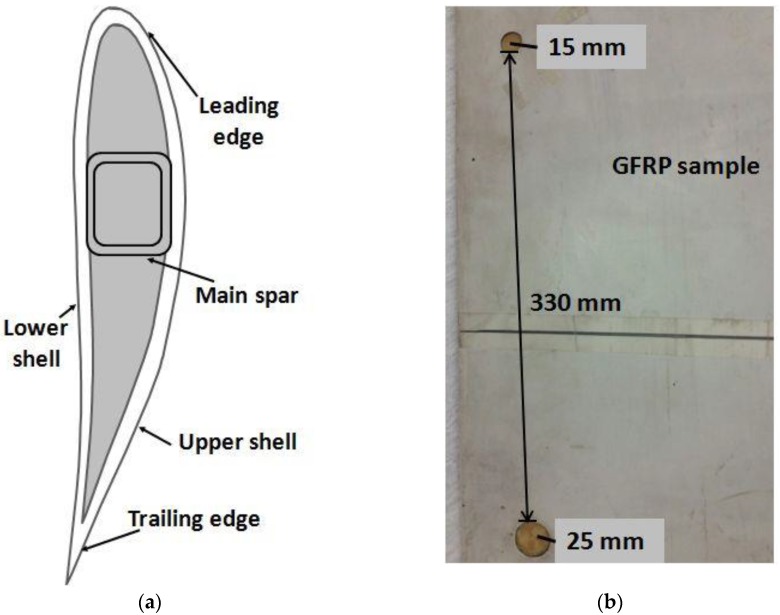
The GFRP sample of the wind turbine blade: (**a**) cross-sectional view of wind turbine blade; (**b**) bottom-side view of GFRP sample showing the defects of 15 mm and 25 mm diameter on the trailing edge of the sample (opposite side of scanning surface).

The sample selected for the experimental analysis was a segment of the wind turbine blade constructed from GFRP composite. The artificially designed disbond type defects with 15 mm and 25 mm diameter present on the trailing edge of the segment were investigated. The end-to-end distance between the defects was 330 mm. The typical cross-section of the sample and its bottom-side view including the defects are shown in Figure 2a and Figure 2b, respectively.

### 3.1. Dispersive Characteristics of the Propagating Wave Modes

**Figure 3:**
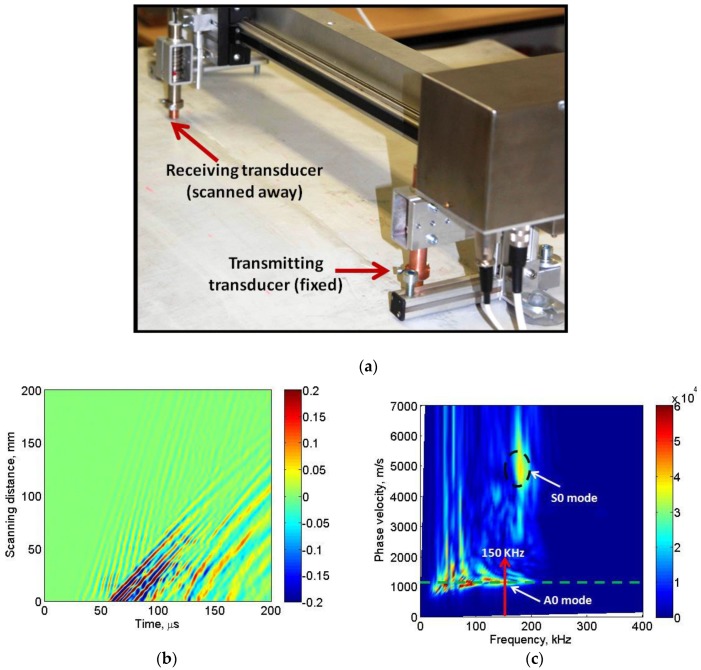
Dispersive characteristics in defect-free region: (**a**) Photograph showing experimental set-up for linear scanning (**b**) the B-scan image; (**c**) dispersion characteristics of phase velocity of propagating modes showing the dominant asymmetric A0 mode and weak S0 mode.

In the first step of the experiment, the dispersive characteristics were estimated in order to find the phase velocity and wavelength of the propagating modes in the defect-free region of the sample. The experiment was performed by keeping the one point-type transducer (transmitter) fixed at a defined point on the sample and another point-type transducer (receiver) scanned away from the transmitter. The initial distance between the transducers was kept at 20 mm. It must be noted that ultrasonic transducers require a protection layer for the testing of composite materials using contact methods. Therefore, wideband ultrasonic transducers with a conical protection layer (diameter of contact surface < 0.3 mm) containing a 6 dB bandwidth up to the 300 kHz had been selected for the experimental analysis [58]. The photograph of the linear-scanning experiment is shown in Figure 3a.

The transmitter was excited using a 150 kHz, three period burst signal with an amplitude of 250 V and the receiver was scanned away up to the scanning distance of 200 mm with a scanning step of 0.5 mm. The resulting B-scan image was registered and later analyzed in MATLAB. The two-dimensional fast Fourier transform (2D-FFT) was applied for post-processing of the resulting B-scan signal in order to obtain the dispersion characteristics of phase velocity of propagating modes [59,60]. All components and the low-frequency ultrasonic system, developed by the Ultrasound Institute, Kaunas University of Technology, were used for the investigation of the sample. A 10-bit analog-to-digital (A/D) converter with sampling frequency 100 MHz was used. The total gain of the measurement system, including external preamplifier and main amplifier, was up to 113 dB. The entire system had two input channels. The USB V2 interface was available to transfer the received, amplified, and digitized ultrasonic signals to the computer for further processing, visualization, and storage. Mechanical scanning of receiving ultrasonic transducer was performed by single axis mechanical scanner having a resolution of 20 μm.

The B-scan image and the dispersion characteristics are shown in Figure 3b and Figure 3c, respectively.

The asymmetric A0 mode and symmetric S0 mode were observed in the dispersion curve as shown in Figure 3b. However, the dominant mode is the A0 mode and its phase velocity at 150 kHz excitation frequency was approximately equal to 1160 m/s. The approximated operating wavelength is therefore equal to 7.7 mm. The phase velocity of the weak S0 mode was approximately within the range of 4500–5500 m/s. The same type of transducers operating in thickness mode will be used in order to scan the defective regions as well in the next step of the experiment. Moreover, the A0 mode would be more sensitive to these types of defects. Hence, it was assumed that the wave mode of the interest is the A0 mode.

### 3.2. Experimental Analysis of the Defective Regions

**Figure 4:**
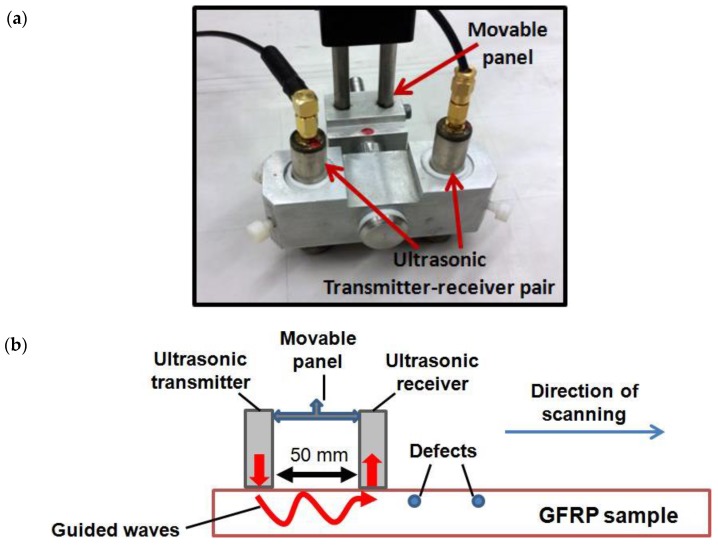

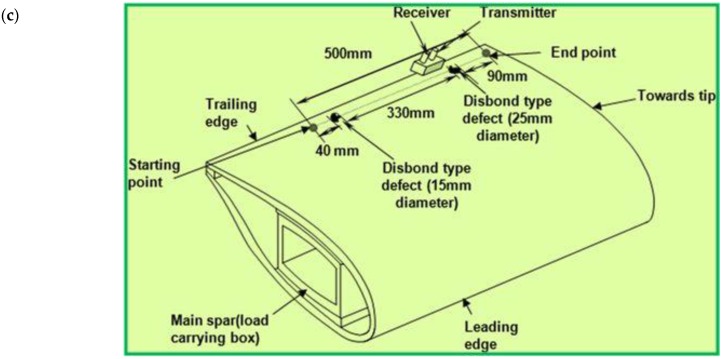
Experimental analysis of disbond type defects in GFRP sample (a segment of WTB): (**a**) photograph of a special arrangement of transmitter–receiver system during the experimental investigation; (**b**) schematic showing the working principle of transmitter–receiver system for GW testing; (**c**) schematic showing the complete arrangement of the scanning process for estimation of 15 mm and 25 mm diameter defects present on the trailing edge of the sample.

In order to perform the ultrasonic GW testing of the GFRP sample (a segment of WTB), a special arrangement of two point-type transducers was developed. The objective of the experiment was to exploit the guided wave properties for the estimation of defects. The special arrangement of the transducer system was developed for the experimental analysis. Two point-type transducers 50 mm apart were mounted on a moving panel and operated in pitch–catch mode. A fixed distance of 50 mm between the transducers was actually equal to few wavelengths of the slowest A0 mode. It also ensured the resolution as required for generation and detection of guided waves after interaction with the sample. Both the transmitter and receiver were acoustically insulated from the casing to avoid any possibility of cross-talk. Both transducers were able to generate multiple modes and had the same characteristics as specified in Section 3.1. The photograph and schematic of transmitter–receiver system for scanning the sample are shown in Figure 4a and Figure 4b, respectively. The major consideration was the interaction of the dominant A0 mode and few other modes with the sample along the propagation path. The basic principle was generation of multiple guided modes by the transmitting transducer and reception by the receiving transducer continuously along the scanning path. The considerable changes in the received waveform would suggest the presence of defects inside the sample.

**Figure 5:**
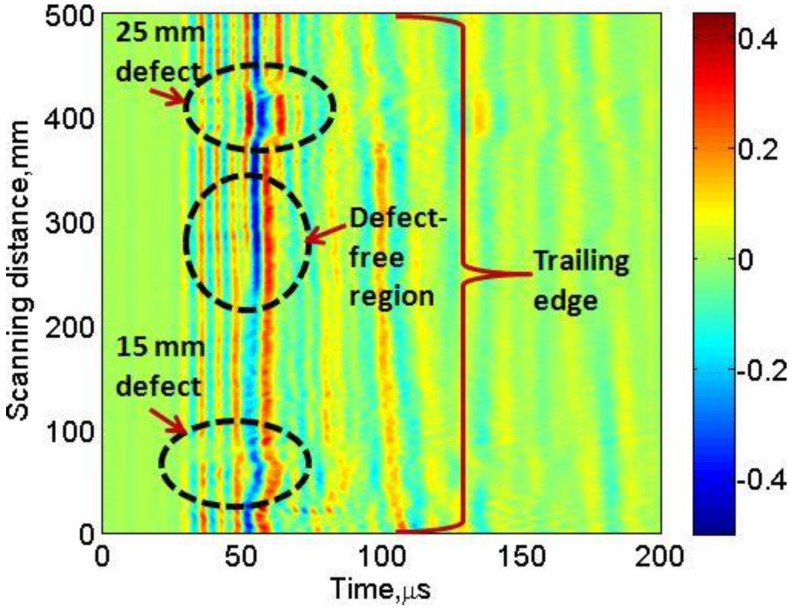
The B-scan image showing the disbond type defects of 15 mm and 25 mm diameter.

The complete arrangement of the experimental analysis of the ultrasonic GW testing of the GFRP sample (WTB) in order to analyze the 15 mm and 25 mm disbond type defects is shown in Figure 4b. The thickness of GFRP sample (WTB) along the trailing edge was 6 mm and both defects were located at 2.5 mm depth from the scanning surface and could not be visually viewed during the experimental investigation. Figure 2b showed only the bottom-side view from the opposite side of the scanning region. The 15 mm defect was distant at 40 mm from the starting scanning point. There was a 330 mm gap between the 15 mm and 25 mm defects and later was presented at 90 mm distance from the end point of scanning. The low-frequency ultrasonic system as described in Section 3.1 was used in the measurement process. The excitation signal was a single pulse of 250 V, 150 kHz. The receiver recorded the 500 A-scans along the scanning distance of 500 mm with the step size of 1 mm. The coupling medium was required to maintain the uniform acoustic contact between the transducers and sample throughout the scanning process. Glycerol was used for this purpose. At each scanning position, the received ultrasonic signals were averaged (number of averaging eight) and stored for further analysis. Figure 5 shows the B-scan image obtained along the scanning distance (0 to 500 mm) with respect to time (0 to 200 μs).

Both types of defects are visible as the waveforms are altered in the defective regions as compared to the defect-free region due to possible mechanisms such as reflections, refractions, mode conversions, etc. The visibility of 25 mm defect is higher in comparison to the 15 mm defect. One of the possible reasons is that bigger defects contain more wavelengths of propagating mode (the A0 mode is more dominant in this case) and the position of 15 mm defect was closer to the starting scanning point. The size and position of defects still cannot be predicted accurately from the B-scan image, therefore, signal processing was required for locating and sizing the defects.

## 4. The Proposed Hybrid Signal Processing Technique

**Figure 6:**
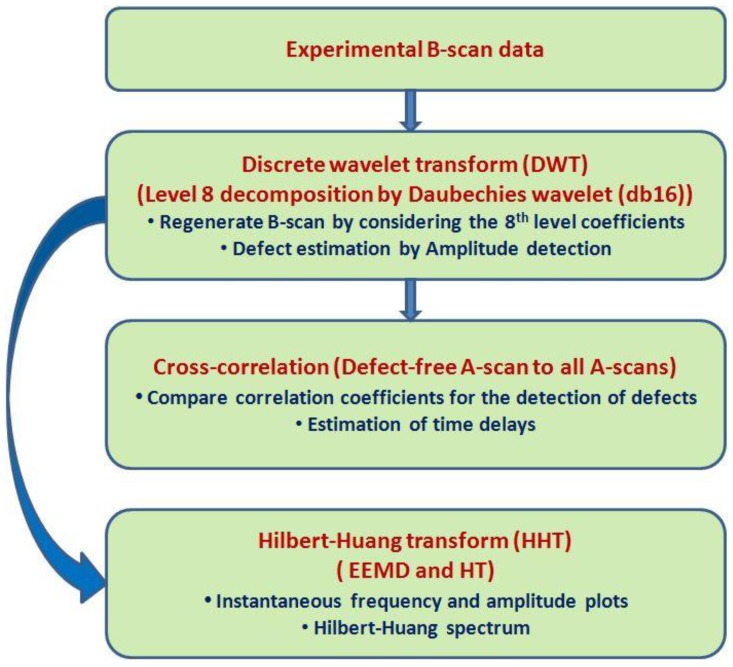
The proposed hybrid signal processing scheme.

In order to improve the SNR and probability of the defect estimation, the scheme of proposed hybrid signal processing algorithm is presented in Figure 6. The proposed signal processing method is a combination of three most promising signal processing techniques, namely, WT, cross-correlation, and HHT as explained in Section 2. The proposed algorithm can be described in the following steps:

Step 1: Wavelet transform
  - Decompose all A-scans of experimental B-scan (Figure 5) into a sum of eight elementary wavelets by DWT using the Daubechies wavelet (db 16).
  - Regenerate the filtered B-scan by using only the eighth level coefficients to minimize the noise.
  - Select the defect-free region by applying proper time-window and estimate the averaged A-scan signal of the selected defect-free region (reference signal).
  - Subtract the reference signal from all A-scans and regenerate the new B-scan image for clear visualization of defects.
  - Compare the reference signal to all A-scans of filtered B-scans by amplitude detection method in order to estimate the size and position of defects.
Step 2: Cross-correlation
  - Find the cross-correlation between the reference signal and all A-scans of wavelet-filtered B-scan along the scanning distance in order to detect the defects by comparing maximum correlation coefficient.
  - Estimate a time delay between the reference signal to each of A-scans along the scanning distance in order to detect the defects by comparing deviations of the time delay.
Step 3: Hilbert–Huang transform
  - Apply proper windowing in the wavelet processed B-scan and find the average value of signals (A-scans) in the selected defect-free and defective regions.
  - Decompose each A-scan into the IMFs using EEMD.
  - Select the appropriate IMFs for the further analysis.
  - Estimate the instantaneous characteristics and Hilbert–Huang spectrum for defect-free and defective signals.

## 5. Results and Discussion

The final results and analysis of defects using the proposed hybrid signal processing are described in the following three subsections.

### 5.1. Defect Analysis Using WT

**Figure 7:**
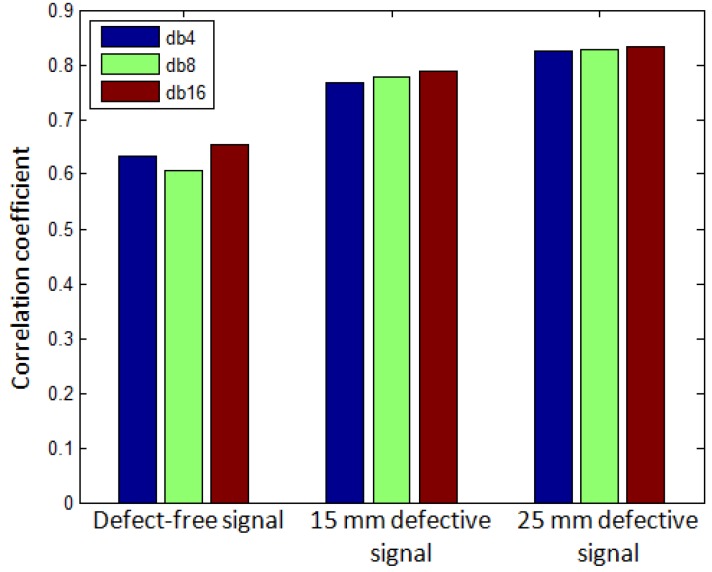
Comparison of maximum correlation coefficients between the 8th level detailed signal by using Daubechies (db4, db8, and db 16) mother wavelet to the original signals.

In the first step, DWT was applied to the experimentally obtained B-scan signal. All resulting A-scans of the experimental B-scan signal were decomposed into the elementary signals which are called wavelets and each wavelet coefficient contained a signal and noise. There was no considerable difference seen by using the different wavelet families but the Daubechies (db) wavelets are the simplest, orthogonal in nature, and more energy preserving [61,62]. The selection of appropriate order of Daubechies (db) wavelet was based on the comparison of maximum value of correlation coefficients between the original signal and the eighth level detailed signals estimated by Daubechies-4 (db4), Daubechies-8 (db8), and Daubechies-16 (db16) mother wavelets in the defect-free and defective regions [63]. The defect-free A-scan signal was estimated by taking the average value within the range of (150–300 mm) in space from the experimental B-scan (Figure 5). Similarly, the A-scan signals in the 15 mm and 25 mm defective regions were estimated by calculating the average value within the space window of (50–60 mm) and (400–410 mm) respectively. Thereafter, detailed signals at level-8 were calculated for each of three signals using the db4, db8, and db16 wavelets. The comparative analysis of maximum cross-correlation between the eighth level detailed signals (wavelet-transformed) and the original signals are illustrated in Figure 7. It can be clearly observed from Figure 7 that the detailed signal obtained with the db16 has a slightly higher correlation in all three cases.

**Figure 8:**
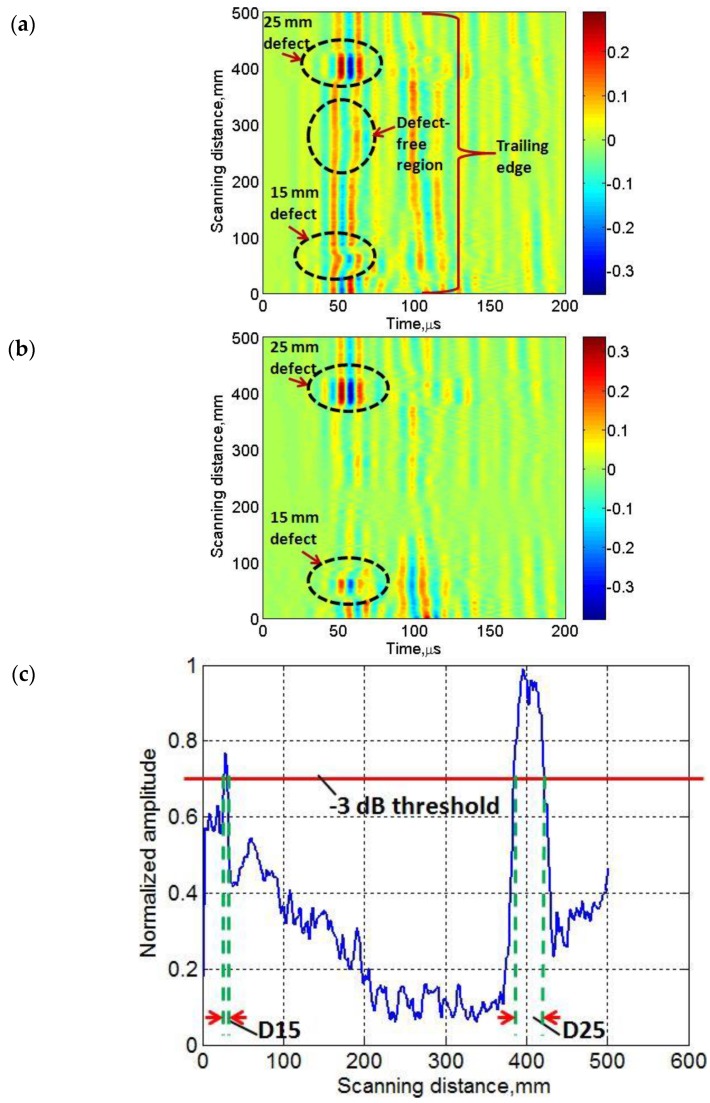
Application of DWT on B-scan signal: (**a**) processed B-scan image by using signals at level-8 by Daubechies wavelet (db16); (**b**) reconstructed B-scan by subtracting the average value (A-scan) of selected defect-free (reference signal); (**c**) detection of both type of defects using amplitude detection by −3 dB threshold (D15 is 15 mm defect and D25 is 25 mm defect).

Therefore, we proposed to use the discrete wavelet transform (DWT) using the Daubechies wavelet (db16) to decompose each A-scan signal into the eight levels. After analysis, it was found that detailed signals of each A-scan at level 8 contain the minimum noise while preserving the signal energy. Finally, the B-scan was regenerated by using all the signals at level 8 as shown in Figure 8a. It can be clearly observed that processed B-scan (Figure 8a) has minimum noise effects as compared to the experimental B-scan (Figure 5).

In the next step, the defect-free region was selected using the proper windowing within the range of (150 mm and 300 mm) in space from the processed B-scan (Figure 8a). The reference A-scan signal (*A*_1, *ref*_) was estimated by taking the average value within the selected defect-free region. The reference signal (*A*_1, *ref*_) was subtracted from all A-scans of the processed B-scan (Figure 8a) and the B-scan image is regenerated again from the resulting signals as shown in Figure 8b. The effect of both defects in Figure 8b is clearly visible and improved. The approximate size and position of defects were analyzed by applying the amplitude detection and comparing the normalized amplitudes of all A-scans of the processed B-scan (Figure 8b). After setting the threshold value of -3 dB (0.7) in this amplitude detection method, both types of defects were easily detected as shown in Figure 8c. The calculated value of lateral dimension using the amplitude detection of 15 mm defect (D15) and 25 mm defect (D25) was 9 mm (percentage error is 40%) and 34.5 mm (percentage error is 38%), respectively. The defect position (center of defect) was calculated as 29 mm (percentage error is 37.5%) and 405.5 mm (percentage error is 2%). It must be noted that error depends not only on the wave dispersive characteristics but also on the uncertainty due to the optimal fixed distance (50 mm) between the transmitting and receiving transducers.

### 5.2. Cross-Correlation on Wavelet-Transformed Signals

As mentioned in Section 2.1 and Section 4, the cross-correlation technique was applied on the wavelet transformed signals (Figure 8a) in order to find the similarities and the time delays between the reference signal to all signals along the scanning distance. As compared to the reference signal *A*_1, *ref*_, the time window was also considered to get the reference signal in the defect-free region. Therefore, the reference region, in this case, was achieved by applying the window function within the range of 20–70 μs in the time domain and within the range of 150–300 mm in the space domain. The time interval was selected on the basis of intensive interaction of propagating waves with the defects in this time interval. The new reference A-scan signal (*A*_2, *ref*_) is then calculated by taking the average value within the selected region. The same time window was also applied to all A-scans of the wavelet processed B-scan (Figure 8a).

The correlation coefficients were then estimated using the cross-correlation between the reference signal (*A*_2, *ref*_) and all signals presented in the wavelet processed B-scan.

**Figure 9:**
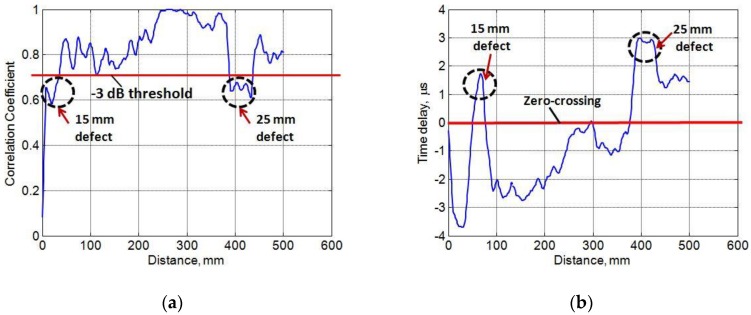
Defect estimation using cross-correlation procedure on wavelet-processed B-scan: (**a**) correlation between the defect-free (reference) signal to all signals; (**b**) estimating time-delays between the defect-free signal to all signals.

The results displayed in Figure 9a shows the variation of the correlation coefficient between the defect-free signal to all signals along the scanning distance. Both defects were detected after applying the −3 dB threshold.

As the total thickness of composite construction reduces in the defective regions, the phase velocity also decreases accordingly. That is why time-delay is an important parameter to distinguish behavior of the guided waves in the defective and defect-free regions. Thus cross-correlation was used again to locate the peak values of delay in order to find the time-delays between the defect-free and defective signals as shown in Figure 8b. The estimated maximum delays were 1.75 μs and 3 μs in the case of 15 mm and 25 mm defects respectively in comparison to the defect-free signal. In both cases, as shown in Figure 9a,b, the moving average filter with eight-point averaging was used.

### 5.3. HHT on Wavelet-Transformed Signals

In the next step of signal processing, the instantaneous characteristics of the defect-free and defective regions were analyzed by applying the HHT to the wavelet-processed signal (Figure 8a). The A-scan signals to be considered for analyses were acquired by applying the proper space-windowing and averaging. These signals are described as follows:

- The defect-free signal was the same as the reference A-scan signal (*A*_1, *ref*_) in the entire time interval.
- The A-scan signal in 15 mm defective region (*A*_15_) was estimated by selecting the space window within the range of (50 mm and 60 mm) and averaging it.
- The A-scan signal in 25 mm defective region (*A*_25_) was estimated by selecting the space window within the range of (400 mm and 410 mm) and averaging it.

**Figure 10:**
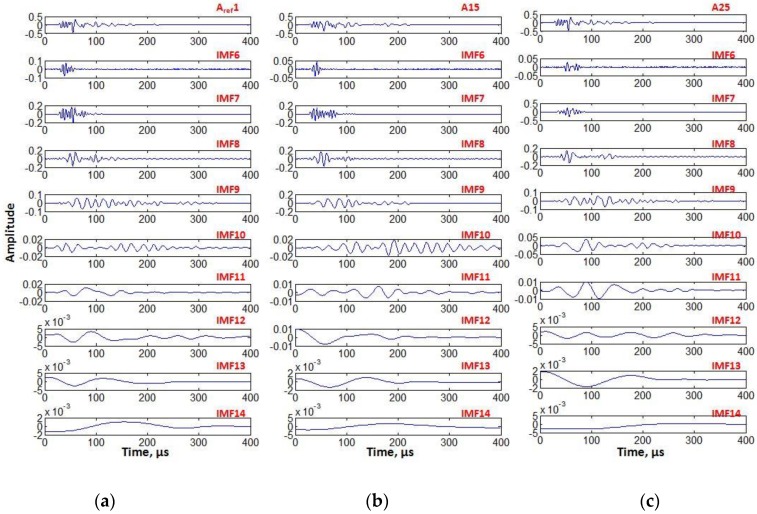
Intrinsic modes by EEMD decomposition: showing the informative intrinsic modes (IMF6–IMF14) of defect-free signal (**a**); 15 mm defective signal (**b**); and 25 mm defective signal (**c**).

Each of three signals was decomposed into the fourteen IMFs (IMF1-IMF14) by EEMD method as described in the Section 2.3.1. The ensemble size (E) of 500 and the noise standard deviation of 0.2 were used throughout the analysis. It was observed that IMF1–IMF5 exhibited in high-frequency regions contained a considerable amount of noise and, therefore, only nine IMFs (IMF6–IMF14) were used for the further analysis of instantaneous characteristics. The defect-free signal (*A*_1, *ref*_), 15 mm defective signal (*A*_15_), and 25 mm defective signal (*A*_25_) with their corresponding IMFs are shown in Figure 10.

**Figure 11:**
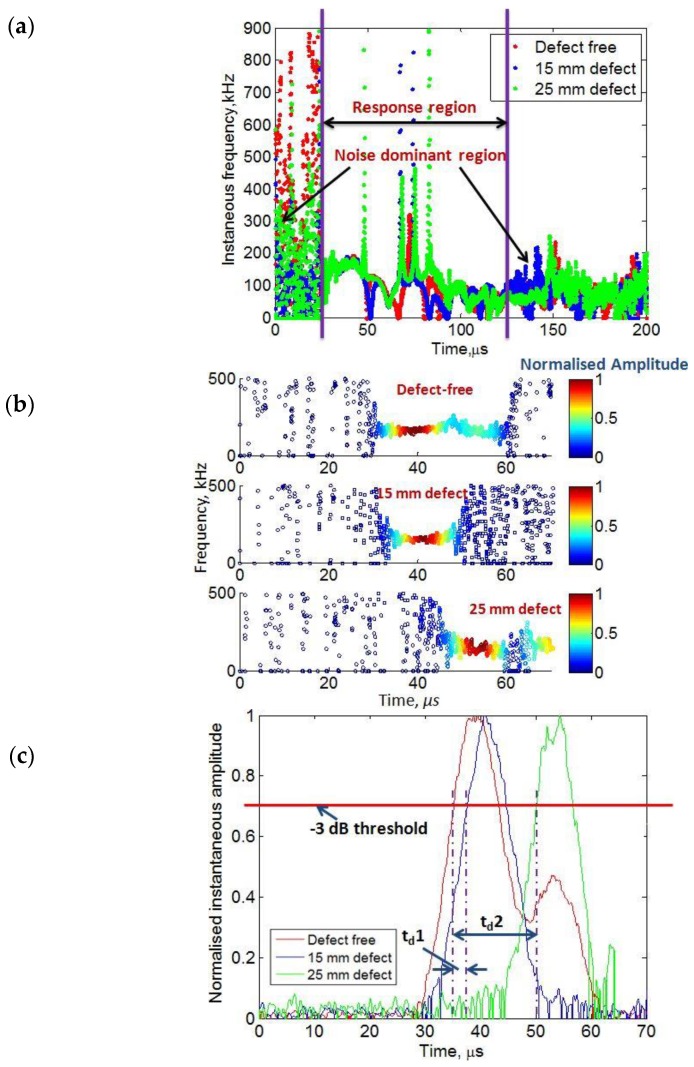
Instantaneous characteristics of defective and defect-free signals using HHT: (**a**) time–frequency characteristics of reconstructed signals (IMF6-IMF12); (**b**) Hilbert–Huang spectrum of IMF6; (**c**) the normalized characteristics of instantaneous maximum amplitude of Hilbert–Huang spectrum of IMF6.

Out of these nine IMFs, the power spectral densities of IMF6–IMF12 were quite similar to their corresponding original time signals. Therefore, IMF6–IMF12 of each signal were added to reconstruct the new signals. The time-frequency characteristics for each of three reconstructed signals were obtained using the HT as described in the Section 2.3.2 and displayed in Figure 11a. The noisy region and response region can be clearly observed. It was observed that oscillations occur around the mean value but the defective signals exhibit increasing modes with higher peak frequencies as the interaction reaches more closely the defective regions in comparison to the defect-free signal.

The time–frequency graph explained the difference among the variation of instantaneous frequencies but it had no information how signal energy varies with respect to time and frequency. In order to show how the instantaneous amplitude of defect-free and defective signals vary with respect to the time and frequency, the HHT spectrum was calculated for all three signals as shown in Figure 11b. The IMF-6 had the highest energy among IMF6–IMF12 and had the most similar power spectral density as of the original signal. Thus, IMF-6 instead of the reconstructed signal was used to calculate the HHT spectrum in the selected interval (0–70 μs).

The reason for the selection of time interval was based on the propagation direction through the damaged region as it covered the time region of interest (20–70 μs). Within this interval, the defects were interacted more intensively with the propagating waves as compared to the other time intervals. HHT spectrum shows the distribution of instantaneous amplitude for each type of signal for the selected IMF in time–frequency axis. The instantaneous frequency is represented in the *y*-axis and the values of amplitude distribution are shown by the color bar. The differences in amplitude distribution between defect-free and defective signals can be clearly observed. Although the amplitude in maximum response region oscillates around the mean frequency of 150 kHz (excitation frequency) for all three cases but the defective signals reached their maximum amplitude (energy) level after the defect-free signal (according to the time-scale). It is also observed from Figure 11b, that defective signals have slightly broader starting time span of low amplitude or noise as compared to the defect-free signal. In the response time interval (0–70 μs), initial noisy time spans were observed as (0–30.65 μs), (0–33.84 μs) and (0–45.30 μs) for the defect-free, 15 mm defective signal, and 25 mm defective signals respectively.

In order to estimate the delay time between the defect-free and defective signals, the normalized maximum instantaneous amplitudes of the Hilbert–Huang spectrum (within the whole frequency bandwidth of ultrasonic transducers) were compared and shown in Figure 11c in the same time interval of (0–70 μs). By selecting the –3 dB threshold, the time delay between the defect-free signal and the 15 mm (*t_d_*_1_) and 25 mm (*t_d_*_2_) defective signals was measured as 2.5 μs and 15 μs, respectively.

## 6. Conclusions

The B-scan image in comparison to the C-scan saves a significant amount of time during the experimental investigation. However, it is a challenging task to size, locate, and compare the defective signals in composite materials from a single B-scan image using only one-side access of a sample by the low-frequency ultrasonic guided waves. The special configuration of point-type transducers fixed on a moving panel was utilized in the presented research to simultaneously record the A-scan signals along the scanning distance in GFRP sample with aerodynamic shape. Thereafter, the novel hybrid signal processing scheme—comprising the wavelet transform, cross-correlation, and Hilbert–Huang transform—was proposed in order to extract the quantitative information of disbond type defects with 15 mm and 25 mm diameters from a single B-scan image. The size and position of the defects were calculated using DWT with the application of Daubechies wavelet (db16) by reducing the considerable noise effects in the experimental B-scan signal. Apart from other parameters, detection of defect size also depends on the half of the operating wavelength (λ/2) and that is why larger defect (25 mm diameter) in comparison to the smaller defect (15 mm diameter) was detected more accurately. The size of defects having diameters 15 mm and 25 mm (at –3 dB threshold level) was measured as 9 mm with a percentage error of 40% and 34.5 mm with a percentage error of 38%. The location of defects at –3 dB threshold level from the start point of scanning was also calculated as 29 mm (for 15 mm defect) with a percentage error of 37.5% and 405.5 mm (for 25 mm defect) with a percentage error of 2%. The amount of error irrespective of the dispersive characteristics of guided waves and mode conversions additionally relies upon the size of defects, the optimal distance between the transmitting and receiving transducers and the excitation frequency. It should be noted that only the single B-scan acquired using the one-side access of a sample was used for the sizing and locating the defects. The amount of error can be reduced by changing the optimal distance between the transducers and excitation frequency and therefore, the effect of excitation frequency and the optimal distance between the transducers on the accuracy of defect size and position can be investigated in future research.

In the next step, cross-correlation technique was also applied first to detect the defects and then time-delays of wavelet-processed signals, acquired over defects having diameters of 15 mm and 25 mm, were measured as 1.75 μs and 3 μs respectively in comparison to the defect-free signal. The feasibility of HHT was also demonstrated to compare the instantaneous characteristics of the guided wave signals in the case of defective and defect-free regions. The instantaneous frequency of the defective signals (IMF6-IMF12) occupied more peaks as it passes through the defective regions of the sample. In order to compare the HHT spectrum and time–amplitude characteristics, the IMF6 containing the maximum power spectral density among (IMF6-IMF12) was selected. The considerable amount of differences was observed among the HHT spectrums of defective and defect-free signals. The noisy time span of the defect-free signal was shorter as compared to the defective signals. By applying the threshold level of –3 dB in time–amplitude characteristics, the difference between time delays of the 15 mm and 25 mm defective signals in comparison to the defect-free signal was clearly observed. The field of the presented research can be extended by including other ultrasonic signal processing methods, such as split-spectrum signal processing and autoregressive moving average spectral analysis, and comparing the results with the proposed hybrid processing scheme.
